# A rare etiology of partial intestinal obstruction and sepsis: idiopathic mesenteric phlebosclerosis

**DOI:** 10.1055/a-2589-0742

**Published:** 2025-05-22

**Authors:** Ben-Hua Wu, Jia-Lin Yuan, Li-Sheng Wang, Wen-Biao Chen

**Affiliations:** 1 12387 Department of Gastroenterology, Shenzhen People’s Hospital, Shenzhen, China; 2 12387 Department of Radiology, Shenzhen Peopleʼs Hospital, Shenzhen, China

A 64-year-old woman presented with abdominal pain and vomiting for 4 days. She had a medical history of long-term use of traditional Chinese medicine prescribed by her family doctor for more than 10 years. Blood biochemistry tests showed an increase in leukocytosis (WBC 17.18 × 10 ^9^ /L, 88% neutrophils) and elevated procalcitonin (76.42 ng/mL), indicating a systemic infection. Notably, noncontrast abdominal CT revealed signs of incomplete bowel obstruction, especially characteristic mesenteric vessel calcification and colonic wall thickening in the cecum ( Fig. 1 ). After empirical treatment with broad-spectrum antibiotics (ertapenem and teicoplanin) for suspected sepsis, the patient’s condition improved, procalcitonin levels returned to normal, and the gastrointestinal function was restored. We then performed a routine colonoscopy and found that the confined colonic mucosa had a dark-purple discoloration, similar to widespread varicosities. When the colon is fully inflated, normal mucosa between the dark purple areas corresponding to the submucosal veins could be observed, which, combined with the patient's previous medication history, clinical manifestations, abdominal CT, and colonoscopy, the patient can be clearly diagnosed with IMP ( Fig. 2 , Video 1 ). After conservative treatment, the patientʼs abdominal pain and vomiting resolved and she tolerated a soft diet. Upon discharge, instructions were given to stop taking traditional Chinese herbal medicines, and polyethylene glycol 4000 was prescribed to maintain bowel regularity.

**Fig. 1:**
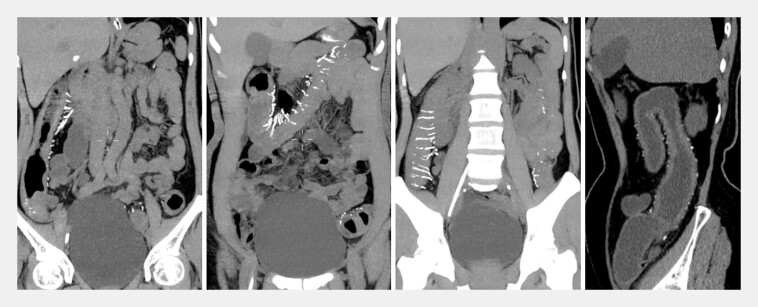
Noncontrast abdominal CT showing extensive calcification and uniform thickening of the colonic wall.

**Fig. 2:**
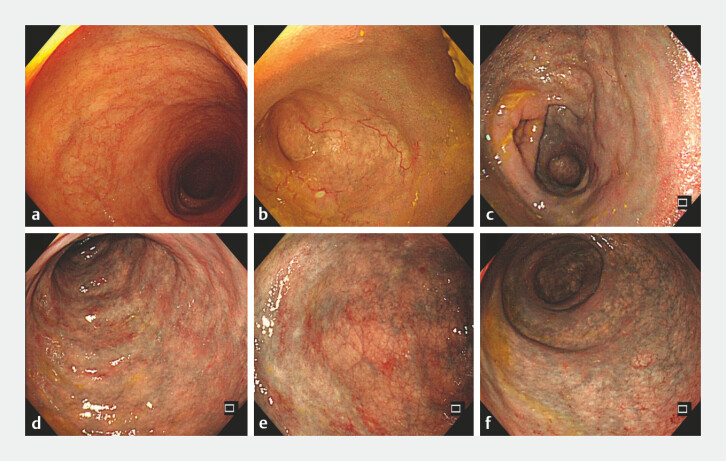
**a** Terminal ileum; **b** cecum; **c** ascending colon; **d** transverse colon; **e** sigmoid colon, closely examined after adequate insufflation; **f** rectum.

This video demonstrates the colonoscopic findings of idiopathic mesenteric phlebosclerosis (IMP), highlighting dark-purple discoloration of the colonic mucosa and normal areas between varicosities.Video 1

IMP is a rare chronic venous disease characterized by mesenteric vein calcification, often associated with the long-term use of Chinese herbal medicines 1 . Diagnosis can be delayed because of the nonspecific presentation 2 3 . Key diagnostic points include long-term use of traditional Chinese medicine, mesenteric vascular calcification on CT imaging, and colonoscopic findings of diffuse dark-purple discoloration of the mucosa, resembling widespread varicosities, with lesions confined to the colon 4 .

Endoscopy_UCTN_Code_CCL_1AD_2AF

## Notes

Endoscopy E-Videos https://eref.thieme.de/e-videos
*E-Videos* is an open access online section of the journal *Endoscopy* , reporting on interesting cases and new techniques in gastroenterological endoscopy. All papers include a high-quality video and are published with a Creative Commons CC-BY license. Endoscopy E-Videos qualify for HINARI discounts and waivers and eligibility is automatically checked during the submission process. We grant 100% waivers to articles whose corresponding authors are based in Group A countries and 50% waivers to those who are based in Group B countries as classified by Research4Life (see: https://www.research4life.org/access/eligibility/ ). This section has its own submission website at https://mc.manuscriptcentral.com/e-videos .
